# Aberrant Somatic Hypermutation at Super-enhancer Drives B Cell Lymphoma Transformation

**DOI:** 10.1093/gpbjnl/qzae015

**Published:** 2024-02-09

**Authors:** Xueshuai Han, Zhaoqi Liu

**Affiliations:** CAS Key Laboratory of Genomic and Precision Medicine, Beijing Institute of Genomics, Chinese Academy of Sciences and China National Center for Bioinformation, Beijing 100101, China; University of Chinese Academy of Sciences, Beijing 100049, China; CAS Key Laboratory of Genomic and Precision Medicine, Beijing Institute of Genomics, Chinese Academy of Sciences and China National Center for Bioinformation, Beijing 100101, China; University of Chinese Academy of Sciences, Beijing 100049, China

## Background

Most B cell lymphomas can be traced back to the germinal center (GC) or B cells that have undergone GC reactions. Within the GC, B cells undergo somatic hypermutation (SHM) and class switch recombination, generating memory B cells or plasma cells with high-affinity antibodies against antigens. Recent studies suggest that the occurrence of activation-induced deaminase (AID)-mediated SHM processes outside the immunoglobulin gene loci may promote the development of lymphomas. This pathological off-targeting of SHM is termed aberrant SHM (aSHM). The DNA targets and potential functional consequences of aSHM are still largely unexplored. Now, through the whole-genome sequencing analysis of longitudinally paired samples representing the transformation from follicular lymphoma (FL) to double-hit lymphoma (DHL, with *MYC* and *BCL2* rearrangements), Uttiya Basu and Jiguang Wang labs have found that the majority of aSHM is distributed around promoter regions embedded within super-enhancers (SEs) [1]. They also reveal that aSHM within SEs can affect the transcription of neighboring genes through enhancer retargeting.

## SHM in normal B cells

AID-mediated deamination of cytidine residues in single-stranded DNA is necessary for SHM of the immunoglobulin variable (IgV) region genes, a process crucial to produce high-affinity antibodies. Genes encoding IgV are actively transcribed in GC B cells, with their DNA released in a single-stranded form by RNA polymerase during transcription, allowing binding with AID. The deamination of cytosine to uracil by AID is the initiating step of SHM. Subsequently, DNA repair is initiated through mismatch repair or base excision repair. This repair process involves error-prone DNA polymerase, such as polymerase eta (Pol η), which tends to preferentially mismatch thymidine regardless of the template sequence. In high-affinity B cells passing through GC reactions, the gene mutation rate at IgV loci is about 1 × 10^6^ times higher than the spontaneous mutation rate in somatic cells. There are roughly nine mutations in each IgV locus, leading to a nearly 100-fold increase in antibody affinity [2,3].

## aSHM in B cell lymphoma transformation

SHM should be rigorously confined to the IgV region genes, with negligible impact on other genomic loci. Nonetheless, in B cell lymphomas originating from GC B cells, aSHM is commonly found in non-coding DNA regions harboring regulatory elements. Recent evidence indicates that non-coding aSHM linked to transcriptional regulatory regions of B cell-specific transcription factors and proto-oncogenes is recurrently observed in diffuse large B cell lymphoma transformed from FL [4]. Despite these observations, the evolution patterns and functional impacts of aSHM in the transformation of lymphomas are not well-explained. Paired longitudinal sequencing of B cell lymphoma samples provides a unique opportunity to address these questions.

Employing whole-genome sequencing analysis on a group of eight patients progressing from FL to DHL, Uttiya Basu and Jiguang Wang labs aimed to delineate aSHM events that occurred at different stages of lymphoma progression, and identify mutations specifically associated with FL transformation [1]. Remarkably, during the transition from FL to DHL, there was a significant increase in mutations around transcription start sites (TSSs) within a 2-kb region. In addition, recurrent copy number gains were observed at the *ZCCHC7*/*PAX5* locus in DHL (6/8). They next assessed the spatial distribution of DHL-associated aSHM. aSHM accumulates notably in SE clusters marked by H3K27ac, which comprise both enhancers and promoters of genes. Further examination of the geographical distribution of transformation-associated aSHM revealed that aSHM was relatively densely distributed near the proximal H3K4me3-marked regions of the promoters of lymphoma-associated genes.

**Figure 1:**
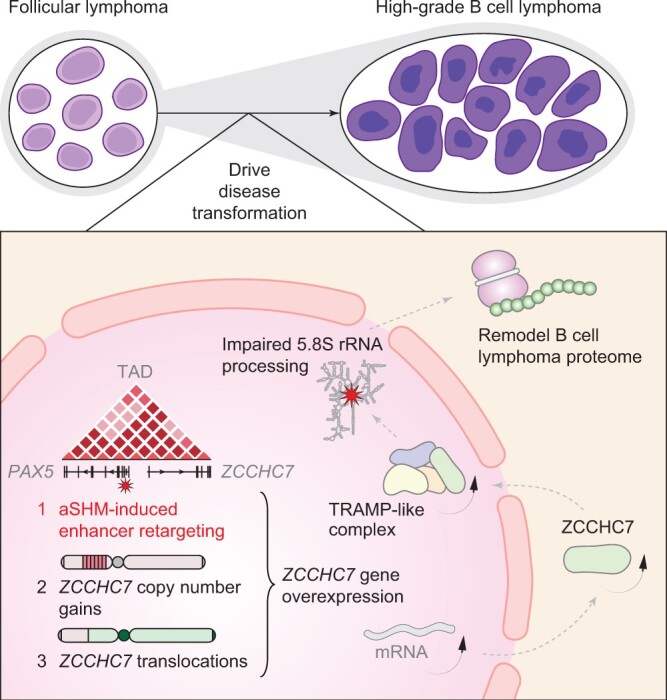
**Schematic overview of the pivotal role of *ZCCHC7* over-expression in lymphoma transformation**The schematic diagram delineates the critical mechanisms implicated in protein synthesis dysregulation during the progression of follicular lymphoma to higher-grade B cell lymphoma. It underscores the role of aSHM in modulating the transcription of the adjacent *ZCCHC7* gene via enhancer retargeting, perturbing canonical 5.8S rRNA processing, thereby culminating in oncogenic alterations within the lymphoma proteome. aSHM, aberrant somatic hypermutation; PAX5, paired box 5; TAD, topologically associated domain; TRAMP, Trf4/5-Air1/2-Mtr4 polyadenylation; ZCCHC7, zinc finger CCHC-type containing 7. The figure was adopted from [1].

Interestingly, they observed an enrichment of transformation-associated aSHM on the alternative TSS of *PAX5* (*PAX5*-TSS2). Different types of genetic alterations were identified in the same region in both diffuse large B cell lymphoma and chronic lymphocytic leukemia. Promoters of *PAX5* and *ZCCHC7* coexist within the same topologically associated domain (TAD) in both mice and humans. Furthermore, a negative correlation in expression between *PAX5* and *ZCCHC7* was noted in a set of diffuse large B cell lymphoma cell lines, a phenomenon that we also observed using data from DepMap portal (https://depmap.org/portal/). These observations suggest that mutations at the *PAX5*-TSS2 may potentially affect the expression of *ZCCHC7* through enhancer retargeting [5] (**Figure 1**). Next, they constructed diffuse large B cell lymphoma cell line models with *PAX5*-TSS2 deletion and point mutations and found that both *PAX5*-TSS2 deletion and point mutations could enhance the interaction between *PAX5* enhancer region and *ZCCHC7* promoter, leading to an increase in *ZCCHC7* mRNA expression without substantial decrease in *PAX5* expression. Moreover, in one DHL patient with elevated *ZCCHC7* expression, a *MYC*-*ZCCHC7* translocation was observed. In summary, *ZCCHC7* was mis-expressed in DHL through three distinct mechanisms: copy number gains, enhancer retargeting, and translocations, implying its oncogenic role in the progression of FL.

Finally, they elucidated cellular functions of *ZCCHC7* and effects of *ZCCHC7* overexpression on lymphoma cells. The AlphaFold prediction model suggests that ZCCHC7 might constitute a component of human Trf4/5-Air1/2-Mtr4 polyadenylation (TRAMP)-like complex, participating in RNA degradation through interaction with exosomes. Increased expression of *ZCCHC7* leads to defects in ribosomal RNA processing, resulting in the accumulation of 5.8S + 40 rRNA. Alterations in rRNA biogenesis lead to aberrant protein synthesis, involving accelerated translation of certain oncogenes and suppression of specific tumor suppressor genes, potentially impacting B cell lymphoma transformation.

## Discussion

Expression of crucial genes determining the B cell lineage, including *BCL6*, *CXCR4*, and *PAX5*, is regulated by nearby SE sequences. Consequently, aSHM within SE clusters may serve as a crucial factor driving the progression of B cell lymphomas. Uttiya Basu and Jiguang Wang labs elucidate the localization of aSHM during lymphoma transformation and demonstrate how hypermutations within SEs alter the transcription of adjacent genes through enhancer retargeting modifications. Additionally, this study also highlights the dysregulation of protein expression in lymphoma due to abnormal rRNA processing in B cell development.

Despite the precise characterization of the target range and potential functional consequences of aSHM in lymphoma progression, mechanisms underlying the generation of pathological aSHM remain unclear. In mouse B cells, aSHM could be induced by the inactivation of mismatch repair or base excision repair genes, while human B cell lymphomas generally lack mutations in these pathways [6]. Intriguingly, AID targets are not randomly distributed throughout the entire genome; instead, they are predominantly concentrated within SEs and regulatory clusters [7]. Future research efforts should aim to elucidate the mechanisms by which AID recognizes DNA target sequences during B cell maturation. Other cellular processes affected by enhancer retargeting in lymphoma cells need to be further explored. Furthermore, beyond enhancer retargeting, it would also be interesting to explore alternative mechanisms through which aSHM in SE clusters influences lymphoma progression. For instance, one recent study reveals that aSHM within *BCL6* SE clusters hinders B-lymphocyte-induced maturation protein 1 (BLIMP1) binding and leads to constitutive expression of *BCL6*. In addition, nuclear receptor subfamily 3 group C member 1(NR3C1) inhibits the expression of *BCL2* and *CXCR4* by binding to their regulatory regions. Interestingly, hypermutated SEs linked to *BCL2* and *CXCR4* prevent the binding of NR3C1 to the regulatory regions of these genes, thus abolishing its inhibitory effect [8].

In conclusion, aSHM within SEs offers a novel perspective for understanding the transformation of B cell lymphomas, providing great potentials for identifying relevant therapeutic targets.

## CRediT author statement

**Xueshuai Han:** Writing – original draft, Writing – review & editing. **Zhaoqi Liu:** Conceptualization, Writing – review & editing, Supervision, Funding acquisition. Both authors have read and approved the final manuscript.

## Competing interests

Both authors have declared no competing interests.
